# Evaluating the effectiveness of integrated care in targeted therapy for patients with chronic lymphocytic leukemia

**DOI:** 10.3389/fonc.2025.1685510

**Published:** 2025-10-21

**Authors:** Xin Liu, Wumin Chen, Meiying Wang, Yan Gao, Xueqin Kou, Xianna Pan, Lijuan Zhang

**Affiliations:** Department of Hematology, Second Medical Center, Chinese People’s Liberation Army General Hospital, Beijing, China

**Keywords:** chronic lymphocytic leukemia (CLL), comprehensive care, targeted therapy, generalized estimating equations (GEE), treatment response

## Abstract

**Background:**

For patients with chronic lymphocytic leukemia (CLL), the type of nursing care during targeted therapy may significantly impact treatment efficacy and quality of life. This study evaluated the effects of routine care and comprehensive care on biomarkers, treatment response, and patient-reported outcome measures (PROMs) in patients with CLL undergoing targeted therapy.

**Methods:**

A total of 260 patients with CLL were enrolled, with 150 receiving routine care and 110 receiving comprehensive care. Baseline characteristics of the two groups were compared, and differences in biomarkers at diagnosis were assessed. Multivariable logistic regression models were used to analyze the impact of baseline information and care methods on treatment response. Generalized estimating equations (GEE) were used to evaluate trends in PROMs (SF-36, ECOG, and HADS scores) over the follow-up period.

**Results:**

Baseline analysis showed no significant differences between the two groups in terms of age, gender, smoking, alcohol consumption, comorbidities, and Rai stage. Drug response analysis showed that age, diabetes, Rai stage and nursing mode significantly affected the treatment effect. Comprehensive nursing significantly increased the effectiveness of targeted drug therapy and reduced the risk of disease progression. During the follow-up period, the lymphocyte count, white blood cell count, ESR, β2-microglobulin, and LDH levels of patients in the comprehensive nursing group significantly decreased, while immunoglobulin levels significantly increased. In addition, comprehensive nursing significantly improves patients’ quality of life, improves physical fitness, reduces anxiety and depression levels, especially in the six months and one year after treatment.

**Conclusion:**

Comprehensive care significantly improved treatment outcomes, reduced disease progression, and enhanced the quality of life in patients with CLL undergoing targeted therapy, making it suitable for long-term management of patients with CLL.

## 1 Introduction

Chronic lymphocytic leukemia (CLL) is a clinically heterogeneous and relatively indolent B-cell malignancy. CLL primarily occurs in middle-aged and elderly populations, with incidence increasing with age. The median age at onset is approximately 70 years, with the highest incidence observed between 60 and 80 years of age (1, 2). Although the disease progresses slowly, the prolonged course of CLL can have a significant impact on patients’ quality of life. As the disease advances, patients with CLL often experience immunodeficiency, recurrent infections, lymphadenopathy, as well as fatigue and decreased physical stamina, which severely affects their daily lives and overall health (3, 4). The purpose of CLL treatment has shifted from the previous requirement of low toxicity and effective reduction of tumor burden to improving the complete remission (CR) rate and minimizing minimal residual disease (MRD) as much as possible. In recent years, due to the continuous development and application of new drugs, prognosis of CLL has greatly improved (5).

Targeted drug therapy is crucial in the treatment of CLL. Its main mechanism involves acting on pathways related to tumor cell growth, reducing the proliferation of CLL cells, and promoting their apoptosis (6). Bruton tyrosine kinase (BTK) inhibitors are commonly used targeted drugs that mainly inhibit the B cell receptor (BCR) signaling pathway, reduce B cell proliferation and survival, and suppress the activity of the PI3K/Akt and MAPK signaling pathways (7, 8). However, the efficacy of targeted drugs is not only related to the drug itself but also closely linked to the patient’s overall health status, such as age, immune function, and comorbidities (9, 10). Therefore, when formulating treatment plans, individual differences of patients need to be considered.

Nursing interventions play a critical role in the management of patients with CLL (11). Nursing care can help alleviate discomfort that may arise during treatment and improve patients’ adherence to treatment as well as their overall quality of life (12). Currently, the primary nursing approaches for patients with CLL include routine care and comprehensive care. Routine care primarily involves basic health education and symptom monitoring to ensure that patients follow their treatment plans and manage adverse reactions promptly. By addressing patients’ physical, psychological, and social needs, comprehensive care not only helps patients better cope with the disease but may also enhance the effectiveness of targeted therapies, potentially providing greater therapeutic benefits. Multiple studies have shown that comprehensive care, through multidimensional interventions including medication management, nutritional support, rehabilitation, psychological counseling, and health education, can improve patients’ treatment adherence, quality of life, and clinical outcomes. For example, comprehensive care has been shown to improve cognitive, physical, and emotional functions, as well as quality of life in patients with lung cancer (13). It has also been reported to alleviate anxiety and depression in patients with liver cancer and reduce the occurrence of complications (14).

Although some studies have shown that nursing interventions can improve the treatment outcomes of patients with CLL, there is still a lack of systematic comparison on the specific effects of different nursing approaches on targeted drug therapy, and there is also a lack of research on the interaction between nursing interventions and time (15). In this context, this study proposes to divide patients with CLL into a routine nursing group and a comprehensive nursing group, observe the effects of the two nursing methods on various outcome indicators of patients during targeted drug therapy, and analyze the interaction between nursing intervention and follow-up time, in order to provide some reference for CLL nursing.

## 2 Materials and methods

### 2.1 Study subjects

This study included 260 patients diagnosed with chronic lymphocytic leukemia (CLL) at our hospital between January 2021 and January 2023, all of whom received targeted drug therapy. All patients signed informed consent forms, and the study was approved by the institutional ethics committee. Inclusion criteria were: 1) Age ≥ 18 years; 2) Received targeted drug therapy; 3) Able to complete a one-year follow-up. Exclusion criteria included: 1) Diagnosis of other malignant tumors; 2) Received other anticancer treatments; 3) Serious mental illness leading to poor compliance; 4) Severe liver or kidney dysfunction.

Among patients who met the inclusion and exclusion criteria, considering that the number of patients receiving routine care in our hospital was much larger than those receiving comprehensive care, we used simple random sampling based on this proportion to select 150 patients from the routine care group and 110 patients from the comprehensive care group for analysis.

### 2.2 Nursing intervention content

Routine nursing mainly includes monitoring vital signs, medication management, and health education.

Comprehensive care: Based on routine nursing, we add medication management, gastrointestinal reaction management, nutritional management, exercise rehabilitation, and psychological support. The nursing staff precisely manages the dosage of targeted therapy drugs to ensure that patients adhere to the prescribed treatment regimen. If patients experience gastrointestinal discomfort such as nausea, vomiting, or diarrhea due to targeted therapy, the nursing staff will collaborate with oncologists to administer antiemetics (such as Ondansetron, which is given intravenously or orally 30 minutes to 1 hour prior to treatment), and provide fluid replenishment (for patients who can eat and do not have severe vomiting, oral rehydration salts are given; if the patient has severe vomiting, diarrhea, or dehydration symptoms, intravenous fluid replenishment will be administered). Nursing staff work with nutritionists to develop a reasonable and comprehensive dietary plan based on the patient’s ability to eat and their condition, primarily focusing on protein and vitamin intake. The typical protein intake is 1.0–1.2 g/kg body weight per day. If the patient is malnourished, the intake can be increased to 1.5–2.0 g/kg body weight, ensuring a total energy intake of at least 25–30 kcal/kg body weight per day. For patients with swallowing difficulties, enteral nutrition support is provided, and plasma, immunoglobulin, and albumin are administered according to medical orders to improve immunity. Nursing staff work with rehabilitation physicians to develop personalized exercise rehabilitation plans, mainly including daily walking and Tai Chi. Walking exercise duration can start from 20 minutes and gradually increase to 30 minutes, depending on the patient’s physical condition. If the patient’s physical condition is poor, segmental exercises can be considered. Tai Chi is performed 3–5 times a week, each session lasting 20–30 minutes. As the patient’s physical condition improves, exercise duration can be gradually extended. For patients who may experience feelings of despair, negativity, anxiety, and depression, nursing staff provide weekly psychological counseling sessions (1–2 times per week) lasting 30–40 minutes with warm, caring words and a kind attitude. In cases of more severe anxiety or depression, collaboration with a psychiatrist will be arranged to help the patient cope with psychological distress.

Each nursing intervention in comprehensive care has clear execution standards, and nursing staff undergo unified professional training. Interventions are personalized based on the patient’s specific needs and treatment plan. All interventions are developed by a professional nursing team, and when the interventions involve specialized areas (such as nutrition, rehabilitation, or psychological health), the nursing team collaborates closely with relevant specialists (such as nutritionists, physical therapists, and psychiatrists) to ensure the comprehensiveness and scientific basis of the interventions.

### 2.3 Data collection

Collect demographic information of patients such as age, gender, smoking, as well as disease-related information such as Rai stage, ECOG score, types of targeted drug, etc. Collect immune-inflammatory indicators at baseline and during follow-up (T1: 1 month, T2: 6 months, T3: 12 months), such as lymphocyte count, white blood cell count (WBC), immunoglobulin G (IgG), immunoglobulin A (IgA), etc. SF-36 score, ECOG score, and HADS score were assessed throughout the follow-up period.

In this study, patients receiving BTK inhibitor therapy were mainly treated with Ibrutinib at a dose of 420 mg orally once daily, continued until disease progression or unacceptable toxicity occurred. Patients receiving BCL-2 inhibitor therapy were mainly treated with Venetoclax, with a weekly dose escalation up to 400 mg orally once daily, taken with food and water. During the study period, all patients received monotherapy, and no combination with other targeted drugs was used.

### 2.4 Data analysis

Three multivariable regression models were constructed in this study. The independent variables included care method, age, sex, comorbidities (such as diabetes and cardiovascular disease), and Rai stage. Model 1: The event was the first achievement of an effective response (complete or partial response) after treatment initiation, with time defined from baseline to event occurrence or end of follow-up. A Cox proportional hazards regression model was used. Model 2: In non-responders, the event was disease progression (PD), with time defined from baseline to progression occurrence. A Cox proportional hazards regression model was used. Model 3: Among responders, the outcome was complete response (CR) versus partial response (PR), analyzed as a binary variable using multivariable logistic regression. IgG, IgA, and IgM levels at 12 months of follow-up were used as dependent variables in a multivariate linear regression analysis. Independent variables included patients’ baseline characteristics (age, gender, smoking, drinking, diabetes, hypertension, cardiovascular disease, and Rai stage) as well as care methods.

To analyze the effects of nursing method and time on PROMs (SF-36, ECOG score, HADS score), a generalized estimating equation (GEE) model was used. This model addresses the correlation in follow-up data and is suitable for repeated measures at different time points. Main effect analysis: evaluating the overall effect of nursing method (routine vs. comprehensive) on SF-36, ECOG score, and HADS score. Time effect analysis: assessing the impact of each time point (T0, T1, T2, T3) on PROMs, evaluating changes in patients’ quality of life, physical functioning, and mental health during follow-up. Interaction effect analysis: analyzing the interaction between nursing method and time, determining whether the nursing method has differentiated effects at various follow-up time points. For example, determining if comprehensive care has more significant effects on improving quality of life and mental health at T2 and T3.

Continuous data were presented as median (min-max) and analyzed using the Wilcoxon test. Categorical data were expressed as frequency (percentage) and analyzed using chi-square or Fisher’s exact test. A two-sided significance level of P < 0.05 was set for all statistical analyses. Data processing and analysis were performed using R software to ensure accuracy and scientific validity.

## 3 Results

### 3.1 Baseline differences in routine care and comprehensive care for patients with chronic lymphocytic leukemia

The results show that the median age of the patients was 60 years, with a range from 42 to 82 years. Among the patients, 149 (57.31%) were male and 111 (42.69%) were female. A total of 94 patients (36.15%) had a history of smoking, and 118 patients (45.38%) had a history of alcohol consumption. The proportion of patients with diabetes was 28.85%, patients with hypertension was 41.92%, and patients with cardiovascular diseases was 58.85%. According to the Rai staging, 35.38% of patients were in Stage 0, 26.54% in Stage I, 19.23% in Stage II, 15.77% in Stage III, and 3.08% in Stage IV. The median SF-36 score for quality of life was 47.39, and the ECOG score showed that 38.46% of patients were in Stage 0. The median HADS score was 10, ranging from 8 to 14. Regarding the use of targeted drugs, 37.31% of patients used Bruton’s tyrosine kinase inhibitors, 25.38% used BCL-2 inhibitors, 11.92% used PI3K inhibitors, and 20.77% used anti-CD20 monoclonal antibodies. 80.38% of patients received first-line treatment, while 19.62% received second-line treatment. Additionally, 12.69% of patients had a history of CLL chemotherapy (**Table 1**).

**Table 1:** Baseline information of patients with chronic lymphocytic leukemia.

Variables	All patients (n=260)	Routine care (n=150)	Comprehensive care (n=110)	P-value
Age	60 (42-82)	60 (42-82)	59 (43-80)	0.979
Gender				0.526
Male	149 (57.31%)	83 (55.33%)	66 (60%)	
Female	111 (42.69%)	67 (44.67%)	44 (40%)	
Smoking				0.050
Yes	94 (36.15%)	62 (41.33%)	32 (29.09%)	
No	166 (63.85%)	88 (58.67%)	78 (70.91%)	
Drinking				0.165
Yes	118 (45.38%)	74 (49.33%)	44 (40%)	
No	142 (54.62%)	76 (50.67%)	66 (60%)	
Diabetes				0.097
Yes	75 (28.85%)	37 (24.67%)	38 (34.55%)	
No	185 (71.15%)	113 (75.33%)	72 (65.45%)	
Hypertension				0.162
Yes	109 (41.92%)	57 (38%)	52 (47.27%)	
No	151 (58.08%)	93 (62%)	58 (52.73%)	
Cardiovascular Disease				0.373
Yes	153 (58.85%)	92 (61.33%)	61 (55.45%)	
No	107 (41.15%)	58 (38.67%)	49 (44.55%)	
Rai Stage				0.780
Stage 0	92 (35.38%)	49 (32.67%)	43 (39.09%)	
Stage I	69 (26.54%)	41 (27.33%)	28 (25.45%)	
Stage II	50 (19.23%)	32 (21.33%)	18 (16.36%)	
Stage III	41 (15.77%)	23 (15.33%)	18 (16.36%)	
Stage IV	8 (3.08%)	5 (3.33%)	3 (2.73%)	
SF-36	47.39 (35.41-58.51)	47.55 (35.69-58.51)	47.23 (35.41-58.31)	0.520
ECOG Score				0.165
0	100 (38.46%)	58 (38.67%)	42 (38.18%)	
1	84 (32.31%)	42 (28%)	42 (38.18%)	
2	58 (22.31%)	40 (26.67%)	18 (16.36%)	
3 or above	18 (6.92%)	10 (6.67%)	8 (7.27%)	
HADS Score	10 (8-14)	10 (8-14)	10 (8-13)	0.813
Types of Targeted Drugs				0.113
Bruton's Tyrosine Kinase Inhibitors	97 (37.31%)	56 (37.33%)	41 (37.27%)	
BCL-2 Inhibitors	66 (25.38%)	31 (20.67%)	35 (31.82%)	
Phosphatidylinositol 3-Kinase Inhibitors	31 (11.92%)	18 (12%)	13 (11.82%)	
Anti-CD20 Monoclonal Antibodies	54 (20.77%)	35 (23.33%)	19 (17.27%)	
Others	12 (4.62%)	10 (6.67%)	2 (1.82%)	
Line of therapy				0.109
First-line	209 (80.38%)	115 (76.67%)	94 (85.45%)	
Second-line	51 (19.62%)	35 (23.33%)	16 (14.55%)	
History of chemotherapy for CLL				0.338
Yes	33 (12.69%)	16 (10.67%)	17 (15.45%)	
No	227 (87.31%)	134 (89.33%)	93 (84.55%)	

### 3.2 Differences in biomarker expression at diagnosis between routine care and comprehensive care groups

Results showed a statistically significant difference in lymphocyte counts between the two groups, with the comprehensive care group having significantly higher counts than the routine care group. There were no significant differences between the two groups in white blood cell count, immunoglobulin G, immunoglobulin A, immunoglobulin M, erythrocyte sedimentation rate, β2-microglobulin, or lactate dehydrogenase (**Table 2**).

**Table 2:** Biomarker levels at diagnosis in patients with chronic lymphocytic leukemia.

Variables	All patients (n=260)	Routine care (n=150)	Comprehensive care (n=110)	P-value
Lymphocyte Count ( /μL)	7828 (5540-10348)	7563 (5540-10215)	8228 (5577-10348)	0.038
White Blood Cell Count (WBC) ( /μL)	53878 (25223-79595)	51620 (25810-78975)	56701 (2522-79595)	0.230
IgG (mg/dL)	575.34 (390.77-759.18)	564.27 (392.03-755.90)	601.35 (390.77-759.18)	0.092
IgA (mg/dL)	48.33 (25.21-67.76)	46.32 (25.21-67.67)	49.62 (26.06-67.76)	0.431
IgM (mg/dL)	26.20 (12.28-40.93)	25.95 (12.73-40.92)	26.30 (12.28-40.93)	0.296
Erythrocyte Sedimentation Rate (ESR) (mm/h)	30.04 (24.05-36.94)	29.77 (24.05-36.94)	30.65 (24.09-36.78)	0.981
Beta-2 Microglobulin (β2-MG) (mg/L)	3.41 (2.20-4.60)	3.35 (2.21-4.60)	3.52 (2.20-4.56)	0.291
Lactate Dehydrogenase (LDH) (U/L)	262.63 (241.27-280.80)	261.24 (241.27-280.80)	264.00 (241.28-280.45)	0.341

### 3.3 Impact of baseline information and care methods on treatment response

In Model 1, age was significantly negatively associated with achieving an effective response (HR = 0.987, 95% CI 0.975–0.999, P = 0.031), indicating that older patients had a slightly lower likelihood of achieving an effective response. Smoking (HR = 0.711, 95% CI 0.525–0.965, P = 0.028) and drinking (HR = 0.696, 95% CI 0.522–0.928, P = 0.013) significantly reduced the probability of achieving an effective response. Care method was positively associated with an effective response (HR = 1.776, 95% CI 1.338–2.359, P < 0.001), with patients receiving comprehensive care more likely to achieve an effective response. In Model 2, age was significantly positively associated with disease progression (HR = 1.034, 95% CI 1.012–1.056, P = 0.002), indicating that older patients had a higher risk of disease progression. Drinking significantly increased the risk of disease progression (HR = 1.882, 95% CI 1.132–3.128, P = 0.015). Care method was significantly negatively associated with disease progression (HR = 0.320, 95% CI 0.169–0.605, P < 0.001), with patients receiving comprehensive care having a substantially lower risk of disease progression. Model 3 analyzed factors influencing complete response within effective responses, showing that smoking had a significant negative correlation with complete response (P < 0.001, OR = 0.659). Rai staging was significantly negatively correlated with complete response (P = 0.030, OR = 0.797); the higher the stage, the lower the likelihood of complete response. Nursing method had a significant positive effect on complete response (P < 0.001, OR = 3.963), with comprehensive care being more likely to achieve complete response (**Table 3**). Multivariate linear regression analysis showed that care methods had a significant positive impact on immunoglobulin levels. Specifically, patients receiving integrated care had significantly higher IgG levels compared with those in the routine care group (β = 9.141, P < 0.001). IgA levels were also significantly increased (β = 1.270, P = 0.001), and IgM levels likewise showed a significant elevation (β = 1.324, P < 0.001), indicating that integrated care was closely associated with overall improvement in immunoglobulin levels (**Supplementary Table 1**).

**Table 3:** The impact of baseline information and care methods on drug response in patients with chronic lymphocytic leukemia.

Term	Estimate	Std.error	Statistic	P.value	HR	CI_lower	CI_upper
Model 1							
Age	-0.013	0.006	-2.158	0.031	0.987	0.975	0.999
Gender	-0.070	0.143	-0.485	0.628	0.933	0.704	1.236
Smoking	-0.341	0.155	-2.191	0.028	0.711	0.525	0.965
Drinking	-0.363	0.147	-2.473	0.013	0.696	0.522	0.928
Diabetes	-0.078	0.162	-0.482	0.630	0.925	0.673	1.270
Hypertension	0.000	0.150	-0.003	0.998	1.000	0.745	1.342
Cardiovascular.Disease	-0.112	0.146	-0.766	0.444	0.894	0.672	1.190
Rai.Stage	0.094	0.062	1.525	0.127	1.099	0.973	1.240
Care Methods	0.575	0.145	3.970	< 0.001	1.776	1.338	2.359

Model 2	Estimate	Std.error	Statistic	P.value	HR	CI_lower	CI_upper
Age	0.033	0.011	3.030	0.002	1.034	1.012	1.056
Gender	0.264	0.259	1.020	0.308	1.302	0.784	2.162
Smoking	0.443	0.257	1.724	0.085	1.557	0.941	2.575
Drinking	0.632	0.259	2.440	0.015	1.882	1.132	3.128
Diabetes	0.131	0.271	0.483	0.629	1.140	0.671	1.938
Hypertension	0.056	0.254	0.220	0.826	1.058	0.642	1.741
Cardiovascular.Disease	0.233	0.260	0.895	0.371	1.262	0.758	2.101
Rai.Stage	-0.125	0.109	-1.144	0.253	0.883	0.713	1.093
Care Methods	-1.140	0.325	-3.506	< 0.001	0.320	0.169	0.605

Model 3	Estimate	Std.error	Statistic	P.value	OR	CI_lower	CI_upper
Age	-0.061	0.016	-3.784	< 0.001	0.941	0.912	0.971
Gender	-0.058	0.357	-0.163	0.871	0.944	0.469	1.899
Smoking	-0.417	0.148	-2.812	< 0.001	0.659	0.463	0.828
Drinking	0.543	0.352	1.542	0.123	1.721	0.863	3.433
Diabetes	0.500	0.369	1.357	0.175	1.649	0.801	3.396
Hypertension	0.205	0.349	0.587	0.557	1.227	0.619	2.432
Cardiovascular.Disease	0.255	0.363	0.704	0.482	1.291	0.634	2.627
Rai.Stage	-0.227	0.152	-1.499	0.134	0.797	0.592	1.073
Care Methods	1.377	0.360	3.828	< 0.001	3.963	1.958	8.022

### 3.4 Changes in inflammatory and cancer markers during follow-up

**Figure 1:**
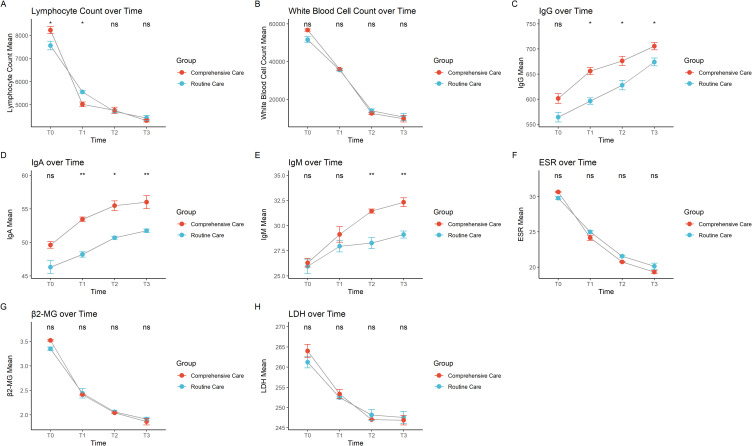
Trends over time in different care groups for **(A)** lymphocyte count, **(B)** total white blood cell count, **(C)** immunoglobulin G, **(D)** immunoglobulin A, **(E)** immunoglobulin M, **(F)** erythrocyte sedimentation rate, **(G)** β2-microglobulin, and **(H)** lactate dehydrogenase. * indicates P < 0.05, ** indicates P < 0.01, and "ns" indicates not significant.

Lymphocyte counts showed a significant decline over time, with the decline slightly greater in the comprehensive care group. White blood cell counts also significantly decreased from T0 to T3, with a similar trend in both groups, but again with a slightly greater decline in the comprehensive care group, suggesting better treatment outcomes in this group (**Figures 1A, B**). Immunoglobulin G, immunoglobulin A, and immunoglobulin M gradually increased over time but did not reach normal levels, and the immunoglobulin levels were higher in the comprehensive care group at each time point compared to the routine care group, suggesting that comprehensive care may be more effective in restoring these immunoglobulin indicators (**Figures 1C–E**). ESR, an indicator of inflammation activity (16, 17), also decreased over time in both groups, with a slightly more pronounced decrease in the comprehensive care group. The faster decrease in the comprehensive care group may be related to its supportive role in reducing inflammation (**Figure 1F**). β2-microglobulin, a marker of tumor burden (18), showed a significant decrease from T0 to T3, with a similar trend in both groups. The slightly greater decrease in the comprehensive care group may indicate that comprehensive care helps reduce tumor burden (**Figure 1G**). LDH, a marker of cell metabolism and damage (19), also decreased, indicating a reduction in tumor cells and cellular damage. The significant decrease in the comprehensive care group suggests that this care method may have a better effect in reducing cellular damage and improving patient metabolism (**Figure 1H**).

### 3.5 Impact of comprehensive care on patient-reported outcome measures

Comprehensive care had a significant positive impact on patient-reported outcome measures. Specifically, it improved SF-36 scores (B = 1.537, SE = 0.289, P < 0.001), indicating enhanced quality of life. It also significantly improved ECOG scores (B = -10.920, SE = 2.349, P < 0.001), reflecting better physical functioning as lower ECOG scores correspond to improved physical condition. Furthermore, comprehensive care reduced HADS scores (B = -5.104, SE = 1.190, P < 0.001), meaning it effectively lowered anxiety and depression levels.

At the first time point (T1), both SF-36 and HADS scores showed significant improvements (P < 0.001), indicating an increase in quality of life and a reduction in anxiety and depression, although ECOG scores did not change significantly (P = 0.078), suggesting limited improvement in physical functioning at this stage. At T2, SF-36 and HADS scores continued to improve significantly (P = 0.011 and P < 0.001, respectively), with noticeable improvements in physical functioning as well (ECOG P = 0.017). By T3, SF-36 approached significance (P = 0.097), indicating stabilization in quality of life, while both ECOG and HADS scores showed significant improvements (P < 0.001), reflecting enhanced physical functioning and reduced anxiety and depression levels.

Interaction effect analysis showed that, at T1, comprehensive care’s impact on SF-36 was not significant (B = 6.127, SE = 4.155, P = 0.140). However, comprehensive care significantly improved ECOG scores (B = -4.417, SE = 1.593, P = 0.006), indicating better physical functioning, and also significantly reduced HADS scores (B = -6.681, SE = 2.442, P = 0.006), meaning a reduction in anxiety and depression levels. At T2, comprehensive care had a significant effect on SF-36 scores (B = 8.151, SE = 3.276, P = 0.013), further improved ECOG scores (B = -7.173, SE = 1.157, P < 0.001), and continued to reduce HADS scores (B = -8.496, SE = 3.907, P = 0.030). By T3, comprehensive care showed a very strong effect on SF-36 (B = 14.273, SE = 5.919, P = 0.016), with the most substantial improvements in ECOG scores (B = -7.415, SE = 1.465, P < 0.001) and HADS scores (B = -6.934, SE = 2.043, P = 0.001), highlighting notable reductions in anxiety and depression (**Table 4**).

**Table 4:** The impact of comprehensive care on patient-reported outcome measures in long-term follow-up.

	SF-36			ECOG score			HADS score		
	B	SE	P-value	B	SE	P-value	B	SE	P-value
Group									
Comprehensive Care	1.537	0.289	< 0.001	-10.920	2.349	< 0.001	-5.104	1.190	< 0.001
Time									
T1	2.898	0.811	< 0.001	-7.987	4.529	0.078	-9.986	4.514	0.027
T2	3.205	1.263	0.011	-4.762	1.992	0.017	-7.266	1.302	< 0.001
T3	5.926	3.567	0.097	-12.952	2.520	< 0.001	-8.905	0.284	< 0.001
Group*Time									
Comprehensive Care*T1	6.127	4.155	0.140	-4.417	1.593	0.006	-6.681	2.442	0.006
Comprehensive Care*T2	8.151	3.276	0.013	-7.173	1.157	< 0.001	-8.496	3.907	0.030
Comprehensive Care*T3	14.273	5.919	0.016	-7.415	1.465	< 0.001	-6.934	2.043	0.001

## 4 Discussion

This study evaluated the impact of regular care versus comprehensive care on treatment outcomes, biomarkers, and patient-reported outcome measures (PROMs) in patients with CLL undergoing targeted therapy. The results show that comprehensive care significantly improves the treatment response rate in patients with CLL, enhances quality of life, alleviates anxiety and depression symptoms, and induces more favorable changes in biomarker levels. These findings provide new scientific evidence to optimize care strategies for patients with CLL, particularly in the context of targeted therapy.

During the follow-up, the levels of immunoglobulins G, A, and M in the comprehensive care group significantly increased and were higher than those in the routine care group. This may be attributed to the nutritional plans included in comprehensive care, which improved patients’ nutritional status, thereby enhancing B cell function and promoting antibody production, leading to higher IgG, IgA, and IgM levels. Medication management ensured the rationality of targeted therapy dosage and infusion rate, reducing damage to organs such as the liver and kidneys, while optimizing treatment efficacy and helping maintain immune function. Psychological support also played an important role by alleviating patients’ anxiety, depression, and stress, thus improving immune function and promoting antibody production. Immunoglobulins are essential defense factors for the body against bacterial, viral, and fungal infections (20). Therefore, elevated immunoglobulin levels suggest that patients’ immune function may be improved, which may help reduce the risk of infections. However, the direct or indirect relationship between increased immunoglobulin levels and a reduced infection rate still requires further investigation in future studies (21). Meanwhile, the decrease in ESR and β2-MG also demonstrates the potential advantages of comprehensive care in reducing inflammation and lowering tumor burden. This indicates that reasonable nutritional management, moderate exercise guidance, and psychological support in comprehensive nursing may jointly promote the recovery of patients’ immune function, thereby enhancing the efficacy of targeted drugs.

The analysis of treatment response indicates that age, diabetes, Rai stage, and care approach are critical factors influencing the treatment response in patients with CLL. Rai stage is also a significant factor, with lower stages associated with a higher likelihood of achieving an effective response. This suggests that early-stage patients with CLL are more likely to exhibit a positive response to targeted therapy, while patients in higher stages with a larger tumor burden and faster disease progression tend to show a relatively poorer response to targeted therapy. Thus, Rai stage could serve as an essential reference for assessing treatment efficacy and prognosis (22). Diabetes also significantly negatively impacts drug response, with patients with diabetes exhibiting poorer treatment outcomes compared to those without diabetes. A possible reason is that patients with diabetes often suffer from immune dysfunction and metabolic disorders, which may weaken the efficacy of targeted therapies (23). Moreover, patients with diabetes may be more prone to vascular complications, which could adversely affect drug distribution and absorption, thereby impacting treatment efficacy. As a significant comorbidity, diabetes warrants special attention in CLL treatment, and incorporating metabolic and glycemic management into the care plan may optimize treatment responses where necessary. Some studies have shown that even in the presence of comorbidities or immune dysfunction, the efficacy of targeted therapies (such as BTK inhibitors or BCL-2 inhibitors) remains favorable, and response rates and progression-free survival are not significantly associated with these factors (24, 25). Therefore, when interpreting the impact of these factors on therapeutic outcomes, it is important to adopt a comprehensive and objective perspective, taking multiple aspects into account, so as to more accurately reflect the real-world treatment responses and prognoses of patients with CLL.

Regarding care strategies, comprehensive care significantly increased the rate of effective response and reduced the risk of disease progression compared to regular care. This demonstrates that, beyond the therapeutic effect of targeted drugs, care interventions play a crucial role in enhancing treatment outcomes. By providing psychological support, nutritional guidance, exercise recommendations, and individualized health education, comprehensive care helps patients better manage the discomforts associated with treatment, improves treatment compliance and tolerance, and ultimately optimizes drug efficacy (26). Notably, across all three models, the significant influence of care approach on treatment outcomes was consistently validated. Especially in terms of complete remission within the effective response group, the comprehensive care group showed a significantly higher rate of complete remission than the regular care group, highlighting the strong positive effect of comprehensive care. This further supports the necessity and value of adopting comprehensive care in managing patients with CLL, as it not only enhances treatment efficacy but also improves quality of life, providing patients with CLL with more comprehensive support.

The results for PROMs also support the advantages of comprehensive care. Quality of life (SF-36 scores) significantly improved, physical condition (ECOG scores) markedly improved, and levels of anxiety and depression (HADS scores) significantly decreased in the comprehensive care group. These improvements were particularly pronounced during the T2 and T3 follow-up stages, suggesting that comprehensive care gradually exhibits its positive effects on quality of life and mental health over the long term. This finding underscores the importance of care interventions in long-term management, particularly for chronic diseases like CLL, where continuous care support can not only enhance patients’ quality of life but also strengthen their ability to cope with the disease.

Richter transformation is a severe complication in the course of CLL, characterized clinically by rapidly enlarging lymph nodes, B symptoms (such as fever, night sweats, and weight loss), and abnormal laboratory parameters, and molecularly by mutations in TP53, NOTCH1, MYC, and complex chromosomal abnormalities, with high aggressiveness and poor prognosis (27). Comprehensive care may help limit or mitigate the molecular aggressiveness of histologic transformation through earlier clinical monitoring and intervention; therefore, future studies should evaluate the role of comprehensive care in the occurrence and early detection of Richter transformation.

Currently, research on nursing in CLL mainly focuses on continuity of care. For example, previous studies have shown that among patients with CLL receiving BTK inhibitor therapy, higher continuity of care was associated with significantly fewer emergency room visits and hospitalizations (28). There are also numerous studies exploring the application of integrated care in cancer patient management. For instance, research has shown that a comprehensive, patient-centered integrated care program for breast cancer patients helps improve long-term survival and quality of life (29). Research on the application of integrated care in CLL is still limited. This study aims to investigate the effects of integrated care interventions on biomarkers, targeted therapy response, quality of life, anxiety and depression, and overall functional status in patients with CLL, with the goal of providing evidence-based guidance for clinical practice and supporting the future implementation of multidisciplinary care models in CLL management.

However, this study has certain limitations. Firstly, as a retrospective study, data selection may involve bias. Although simple random sampling was applied to reduce selection bias, the study was limited by its sample size and single-center design, and unmeasured confounding factors may still have influenced the results. Secondly, the follow-up period was relatively short, making it difficult to comprehensively observe the long-term effects of care interventions. Future studies should consider longer follow-up periods to evaluate the impact of comprehensive care on the long-term prognosis of patients with CLL. Additionally, this study focused primarily on care approaches during targeted therapy without in-depth analysis of the effects of different types of targeted drugs. Future research could explore the synergistic effects of specific targeted drugs and care approaches. Other potential influencing factors, such as geriatric assessment scores, were not fully incorporated. Future studies should consider including more elderly-specific assessment tools (e.g., Comprehensive Geriatric Assessment [CGA], Frailty Index, or G8 score) to further investigate the impact of nursing interventions on treatment outcomes and quality of life in patients with CLL.

## 5 Conclusion

In summary, this study indicates that comprehensive nursing can improve the treatment response rate, enhance biomarker levels, and improve the quality of life and mental health of patients with CLL in targeted drug therapy. Therefore, it is recommended to include comprehensive nursing interventions in the treatment plan for CLL in order to achieve better clinical outcomes. Future research can further explore the mechanisms of comprehensive nursing and its potential application in different treatment strategies, providing more scientific basis for the management of patients with CLL.

## Data Availability

The raw data supporting the conclusions of this article will be made available by the authors, without undue reservation.
