# Supplementary material for: Evaluating the effectiveness of integrated care in targeted therapy for patients with chronic lymphocytic leukemia
Source: Front Oncol. 2025 Oct 21;15:1685510. doi: 10.3389/fonc.2025.1685510 (PMC12582956; doi:10.3389/fonc.2025.1685510)
Supplement: Supplementary file 1 [file Table1.docx]

| **Supplementary Table. 1 The impact of care methods and baseline characteristics on immunoglobulin levels** | | | | |
| --- | --- | --- | --- | --- |
| **Term** | **estimate** | **std.error** | **statistic** | **p.value** |
| **IgG** |  |  |  |  |
| **Age** | -0.157 | 0.100 | -1.567 | 0.118 |
| **Gender** | 0.451 | 2.375 | 0.190 | 0.850 |
| **Smoking** | -2.665 | 2.462 | -1.083 | 0.280 |
| **Drinking** | -5.849 | 2.375 | -2.463 | 0.014 |
| **Diabetes** | 1.726 | 2.601 | 0.663 | 0.508 |
| **Hypertension** | 4.729 | 2.412 | 1.961 | 0.051 |
| **Cardiovascular.Disease** | 0.710 | 2.402 | 0.296 | 0.768 |
| **Rai.Stage** | 0.281 | 0.991 | 0.283 | 0.777 |
| **Care Methods** | 9.141 | 2.391 | 3.823 | < 0.001 |
| **IgA** |  |  |  |  |
| **Age** | -0.035 | 0.016 | -2.187 | 0.030 |
| **Gender** | 0.294 | 0.384 | 0.766 | 0.444 |
| **Smoking** | 0.214 | 0.398 | 0.538 | 0.591 |
| **Drinking** | -0.075 | 0.384 | -0.194 | 0.846 |
| **Diabetes** | -0.109 | 0.421 | -0.259 | 0.796 |
| **Hypertension** | 0.257 | 0.390 | 0.658 | 0.511 |
| **Cardiovascular.Disease** | -1.068 | 0.389 | -2.747 | 0.006 |
| **Rai.Stage** | -0.177 | 0.160 | -1.103 | 0.271 |
| **Care Methods** | 1.270 | 0.387 | 3.283 | 0.001 |
| **IgM** |  |  |  |  |
| **Age** | -0.015 | 0.013 | -1.191 | 0.235 |
| **Gender** | -0.072 | 0.307 | -0.235 | 0.814 |
| **Smoking** | 0.572 | 0.318 | 1.796 | 0.074 |
| **Drinking** | -0.619 | 0.307 | -2.015 | 0.045 |
| **Diabetes** | 0.609 | 0.336 | 1.811 | 0.071 |
| **Hypertension** | -0.636 | 0.312 | -2.038 | 0.043 |
| **Cardiovascular.Disease** | -0.911 | 0.311 | -2.934 | 0.004 |
| **Rai.Stage** | -0.258 | 0.128 | -2.011 | 0.045 |
| **Care Methods** | 1.324 | 0.309 | 4.280 | < 0.001 |
